# Facile Fabrication of NIR-Responsive Alginate/CMC Hydrogels Derived through IEDDA Click Chemistry for Photothermal–Photodynamic Anti-Tumor Therapy

**DOI:** 10.3390/gels9120961

**Published:** 2023-12-07

**Authors:** Ali Rizwan, Israr Ali, Sung-Han Jo, Trung Thang Vu, Yeong-Soon Gal, Yong Hyun Kim, Sang-Hyug Park, Kwon Taek Lim

**Affiliations:** 1 Department of Smart Green Technology Engineering, Pukyong National University, Busan 48513, Republic of Korea; arizwan92@outlook.com (A.R.); israrchem@gmail.com (I.A.); vutrungthang29@gmail.com (T.T.V.); yhkim113@pknu.ac.kr (Y.H.K.); 2 Industry 4.0 Convergence Bionics Engineering, Pukyong National University, Busan 48513, Republic of Korea; josunghan91@gmail.com; 3 Department of Fire Safety, Kyungil University, Gyeongsan 38428, Republic of Korea; ysgal@kiu.ac.kr; 4 Major of Display Semiconductor Engineering, Pukyong National University, Busan 48513, Republic of Korea; 5 Institute of Display Semiconductor Technology, Pukyong National University, Busan 48513, Republic of Korea

**Keywords:** carboxymethyl cellulose-norbornene, alginate-methyl tetrazine, indocyanine green, NIR-responsive hydrogels

## Abstract

Novel chemically cross-linked hydrogels derived from carboxymethyl cellulose (CMC) and alginate (Alg) were prepared through the utilization of the norbornene (Nb)–methyl tetrazine (mTz) click reaction. The hydrogels were designed to generate reactive oxygen species (ROS) from an NIR dye, indocyanine green (ICG), for combined photothermal and photodynamic therapy (PTT/PDT). The cross-linking reaction between Nb and mTz moieties occurred via an inverse electron-demand Diels–Alder chemistry under physiological conditions avoiding the need for a catalyst. The resulting hydrogels exhibited viscoelastic properties (G′ ~ 492–270 Pa) and high porosity. The hydrogels were found to be injectable with tunable mechanical characteristics. The ROS production from the ICG-encapsulated hydrogels was confirmed by DPBF assays, indicating a photodynamic effect (with NIR irradiation at 1–2 W for 5–15 min). The temperature of the ICG-loaded hydrogels also increased upon the NIR irradiation to eradicate tumor cells photothermally. In vitro cytocompatibility assessments revealed the non-toxic nature of CMC–Nb and Alg–mTz towards HEK-293 cells. Furthermore, the ICG-loaded hydrogels effectively inhibited the metabolic activity of Hela cells after NIR exposure.

## 1. Introduction

Malignant tumors have continued to be one of the major threats to human health, closely related with a high mortality and recurrence rate [1]. In order to cure the cancer cells, doxorubicin (DOX) has been prominently used for various types of liver, breast [2], and colon tumors [3]. However, cardiotoxicity, the primary side effect of DOX which could occur both acutely and persistently and impact patients’ general health, prevented clinical application of DOX [4]. To address this issue, researchers have been adopting photothermal/photodynamic (PTT/PDT) strategies for cancer treatment, reducing patient suffering [5]. This technique represents a noninvasive therapy inducing the generation of reactive oxygen species (ROS) as well as the rise of temperature by irradiating near-infrared (NIR-808 nm) light to kill cancer cells [6]. The development of sophisticated delivery systems is essential in the effort to enhance the effectiveness of PTT/PDT. Among these, polysaccharide-derived hydrogels have garnered significant attention owing to their excellent biocompatibility and biodegradability, initiating no immune reaction, allowing polysaccharide to be an ideal candidate for a hydrogel scaffold [7]. These biocompatible hydrogels have been extensively applied in numerous biomedical areas such as tissue engineering [8], drug delivery [9], 3D bio-printing [10], and wound healing [8,11].

Concerning this, naturally occurring polysaccharides such as chitosan, alginate (Alg), guar gum, hyaluronic acid and carboxymethyl cellulose (CMC), which have extended chains of mono/disaccharide linked by glyosidic bonds, are thought to be handy biopolymers [12]. Among them, Alg, which has excellent biocompatibility and gel-forming properties, is utilized as an economical source of biomaterials extracted from seaweed [13]. On the other hand, the high hydrophilicity, low viscosity and facile modification capability of CMC makes it a suitable candidate for developing biocompatible hydrogels [14]. Combining these two biopolymers, Alg/CMC, can offer a unique opportunity to exploit their synergistic properties for the development of hydrogels for PTT/PDT applications.

A major obstacle in fabricating hydrogels from polysaccharides was regarded to be the requirement for an effective and facile fabrication method. Historically, Alg has been cross-linked using ionic cross-linking, which resulted in poor mechanical properties, thus limiting its capacity to load drugs [15,16,17]. The utilization of the -COOH functional group within Alg polymer chains could simplify the process of functionalizing Alg. As a result, a wide range of covalent cross-linking methods, such as the thiol–ene reaction [18], azide alkyne cycloaddition [19], and photochemical cross-linking [20] has been used for development of hydrogel systems. Although these approaches were simpler and more practical, the majority of them contained hazardous chemicals and catalysts that could have a detrimental impact on associated biopolymers. Hence, there was a need for methodologies that reduced adverse effects when working with polysaccharide-derived hydrogels. Recently, “click” reactions have emerged as a successful alternative approach to produce Alg hydrogels with minimal toxicity [21]. The inverse electron-demand Diels–Alder reaction (IEDDA) reaction provided a high degree of chemo-selectivity and boasted an exceptionally rapid reaction rate under mild conditions. Furthermore, it generated N_2_ gas as a by-product, which could serve as a pore-forming agent [22]. Hydrogels featuring porous structures could offer added benefits when it comes to drug loading and release characteristics. However, the majority of the techniques described in the literature have utilized harmful co-solvents such as DMSO in the fabrication process of hydrogel networks, potentially compromising their biocompatibility [23].

When designing therapeutic carriers for controlled drug release, another crucial factor to consider is the responsiveness of the polymeric hydrogels to external stimuli. These stimulus-responsive hydrogel systems can be triggered by various external stimuli, including pH, temperature, redox reactions, near-infrared (NIR) light, ultrasound, and magnetic fields. Among these stimuli, NIR light is considered safer for cells due to its low radiation energy (in the range of 650–900 nm) and its ability to penetrate tissues effectively, thereby inducing a biochemical response [24]. Our research group has incorporated indocyanine green (ICG), a photosensitizer and NIR-responsive dye, into polysaccharide-derived hydrogels for NIR-responsive drug delivery applications [25]. ICG possesses the capability to generate ROS through the absorption of near-infrared (NIR) irradiation at 808 nm [26]. ROS could stimulate the degradation of hydrogels and on-demand release of therapeutic agents in a controlled manner. On the other hand, ROS itself can be useful for PDT [27]. It is also known that NIR absorption to ICG generates heat as well as ROS, which is utilized in PTT [28].

**Scheme 1:**
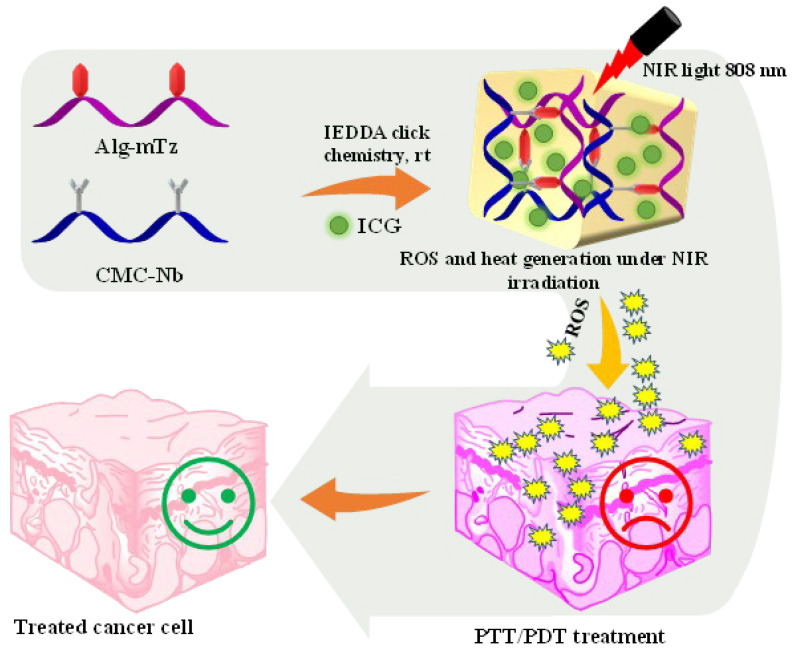
The schematic of CMC/Alg-derived hydrogels and the mechanistic pathway for ROS and heat generation under irradiation of NIR laser (808 nm).

In the current research, we have designed a facile injectable hydrogel system having the ability to produce ROS. The injectable CMC/Alg hydrogel was depicted in Scheme 1, based on the IEDDA click cross-linking reaction between Alg–methyl tetrazine (Alg–mTz) and CMC–norbornene (CMC–Nb). ICG was incorporated during the formation of hydrogels, which could produce ROS with elevation in temperature upon NIR irradiation. In the cell line under investigation, CMC–Nb, Alg–mTz, and the fabricated hydrogels displayed no cytotoxic effects. In contrast, the ICG-loaded hydrogels showed anti-tumor properties against the HeLa cell line and inhibited the explosion of HeLa tumor cells upon exposure to NIR light.

## 2. Results and Discussion

### 2.1. Synthesis of CMC–Nb

**Scheme 2:**
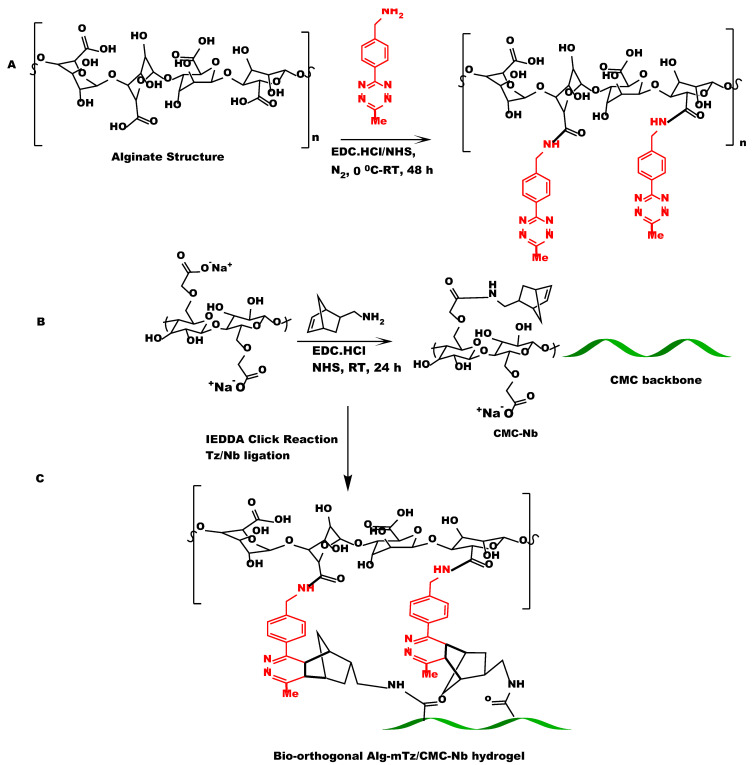
The synthetic route path for CMC–Alg conjugation, (**A**) bio-conjugation of Alg with methyl tetrazine amine, (**B**) functionalization of CMC with norbornene amine, and (**C**) formation of Alg/CMC-derived click hydrogels.

**Figure 1:**
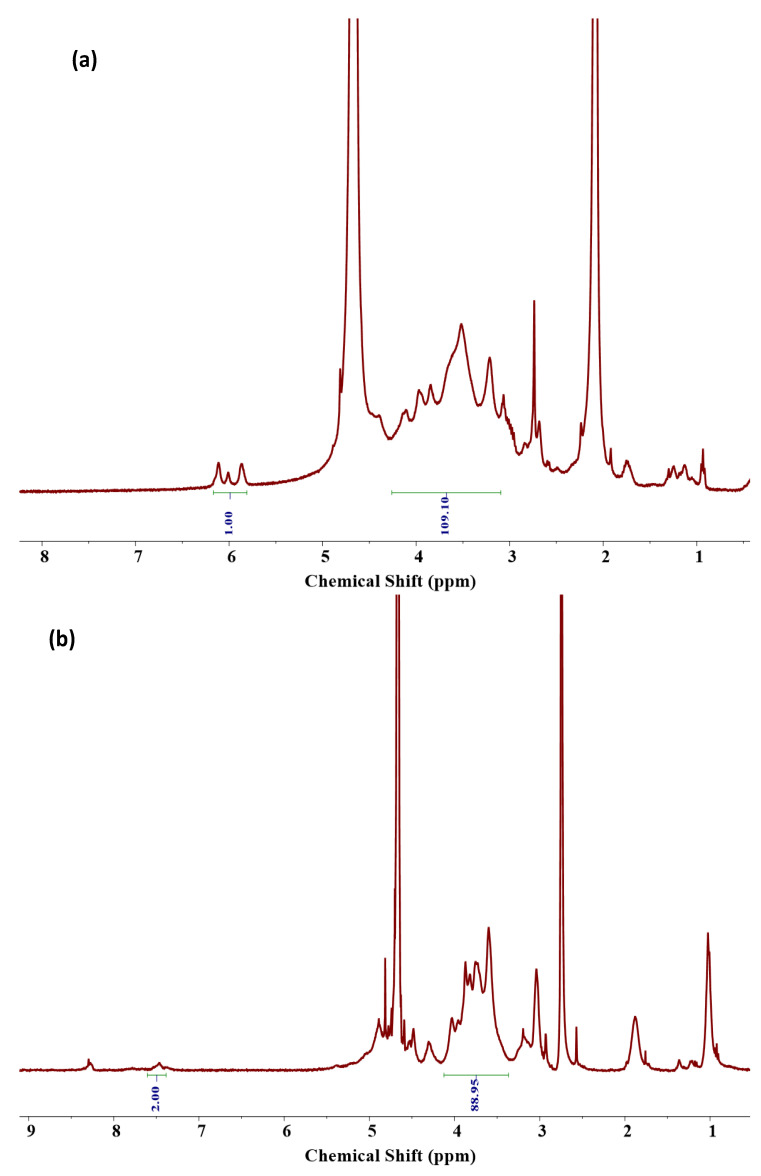
^1^H-NMR spectra of the precursors. (**a**) CMC–Nb and (**b**) Alg–mTz.

The synthesis of CMC–Nb was carried out with 5-norbornene-2-methyl amine and CMC by the EDC/NHS coupling reaction (Scheme 2B). The conjugated CMC was purified by acetone followed by dialysis against a 14 KDa membrane using DI water. The norbornene functional moieties provided reactive sites for mTz counter groups located at the backbone of the Alg polymer to perform an IEDDA click reaction for the formulation of CMC/Alg hydrogels. The 1H-NMR spectrum verified the incorporation of Nb into the CMC backbone, as illustrated in Figure 1a. The double bond signals of the Nb groups were observed in the 5.9–6.2 ppm range, while the region between 3.2–4.5 ppm corresponded to the CMC backbone [29]. The degree of substitution (DS), which was calculated to be 7.7%, was determined by integrating the protons associated with Nb and the CMC backbone.

### 2.2. Coupling of Alg with Methyl Tetrazine Amine

Alg was conjugated by mTz using the EDC/NHS coupling mechanism through which carboxylic functional groups of the Alg molecule were allowed to react with methyl tetrazine amine to form Alg–mTz presented in Scheme 2A. The modification of Alg with mTz, as depicted in Figure 1b, was verified through the 1H-NMR spectrum. The peaks appeared at 7.5 ppm and 8.5 ppm corresponding to the benzene ring proton of the tetrazine ring, which is evidence of the conjugation of Alg with methyl tetrazine amine [30]. DS was determined to be 3.2%, calculated by the ratio of protons from the benzene ring to the backbone of the Alg molecule.

### 2.3. Preparation and Mechanical Properties of CMC/Alg-Derived Hydrogels

Two different kinds (CA-1 and CA-2) of CMC/Alg-derived hydrogels were fabricated with varying Nb/Tz molar feed ratios (Table 1). After agitating the precursor solutions by using a vortex mixer, the Nb and mTz moieties were engaged in an IEDDA click reaction that produced CMC/Alg-derived hydrogels. The optical images of fabricated hydrogels were shown in Figure S1. The interaction between the Nb and mTz groups was confirmed by analyzing the FT-IR spectra of the hydrogel samples.

**Figure 2:**
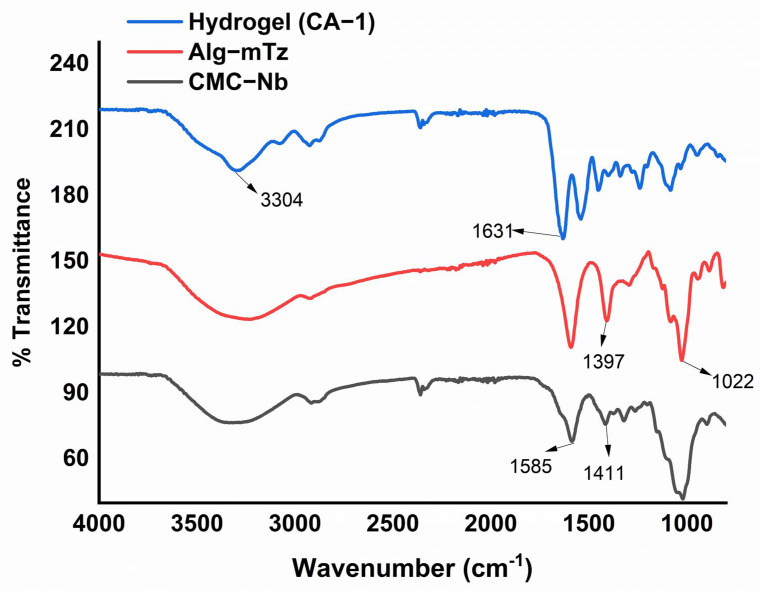
FT-IR spectra of CMC–Nb, Alg–mTz and formulated hydrogels.

The FT-IR spectra (Figure 2) displayed the characteristic alkene bond peak (C=C) of mTz at 1631 cm^−1^, indicating the successful introduction of mTz moieties through the IEDDA click reaction. The C-H peak of the methyl group which appeared at 1397 cm^−1^ gave indication of the methyl (-CH_3_) attached to the benzene ring of the tetrazine moiety. According to the molar concentrations of Alg–mTz in the reaction formulation, the hydrogels were assigned to CA-1 and CA-2. The concentration of the Alg–mTz solution could alter the cross-linking density. The rapid IEDDA click reaction resulted in effective conversion without the need for a catalyst [31].

**Figure 3:**
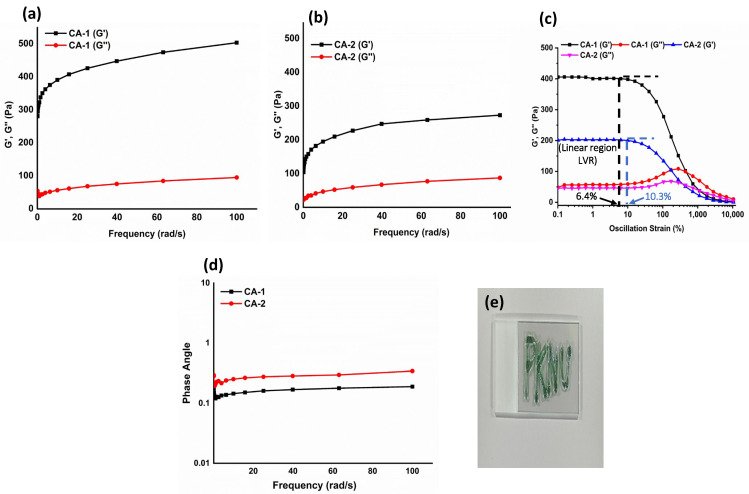
The viscoelastic characteristics of the hydrogels, with (**a**,**b**) representing the moduli as a function of angular frequency of CA-1 and CA-2 hydrogels, respectively, (**c**) the strain amplitude sweep experiment, (**d**) phase angle as a function of angular frequency, (**e**) photographs of injectable hydrogels loaded with ICG.

The rheological characteristics of hydrogels are crucial for drug delivery applications [32]. We measured the mechanical robustness of the CMC/Alg-derived hydrogels by computing the storage/loss modulus (G′/G″). The acquired values were subsequently graphed against angular frequency, as shown in Figure 3a,b.

In the frequency sweep analyses, G′ consistently exhibited higher values than G″, elucidating the flexible nature of the fabricated hydrogels. It could be observed that the G′ of the hydrogels increased as the feed ratios of the tetrazine counterpart increased (which resulted in higher cross-linking density). The storage modulus values at the initial frequency of the hydrogels (CA-1 and CA-2) were measured as 492 and 270 Pa, as depicted in Figure 3a,b, respectively. These outcomes show that the mechanical robustness of hydrogels has a direct relation with the extent of cross-linking as described previously [33].

The viscoelastic nature of the hydrogels was further investigated by analyzing dynamic oscillatory strain amplitude-sweep tests. Precisely, the study was conducted to confirm the linear viscoelastic region (LVR) of hydrogels as presented in Figure 3c. The viscoelastic behavior of hydrogels, characterized by their G′ and G″ values, remains relatively consistent regardless of the level of applied strain. The hydrogels CA-1 and CA-2 exhibited LVR within the respective ranges of 0.1–6.4% and 0.1–10.3% respectively, demonstrating that sufficient cross-linking leads to the decreased value in LVR of hydrogels.

It is also reported in the literature that LVR steadily decreases as a hydrogel turns into a solid phase [34]. The critical strain (γc%), which can be defined as the point at which G′ values deviate by more than 5% from the plateau values, is a key parameter for hydrogels [35]. The critical strains were observed at various strain amplitudes (15% and 25%), for CA-1 and CA-2, respectively. Beyond the γc%, the values of G′ gradually diminish, indicating the transition of hydrogels from a semi-solid state to a semi-liquid state. Additionally, the phase angle (<1) of hydrogels was assessed across an angular frequency (0.1–100 rad/s), indicating that hydrogels were elastic [36] as presented in Figure 3d. The injectability of the hydrogels was evaluated by passing CA-1 through a 25-gauge needle, as shown in Figure 3e, which confirmed that the hydrogels could be precisely delivered into deteriorated tissues or organs.

### 2.4. Swelling Performance of Hydrogels

**Figure 4:**
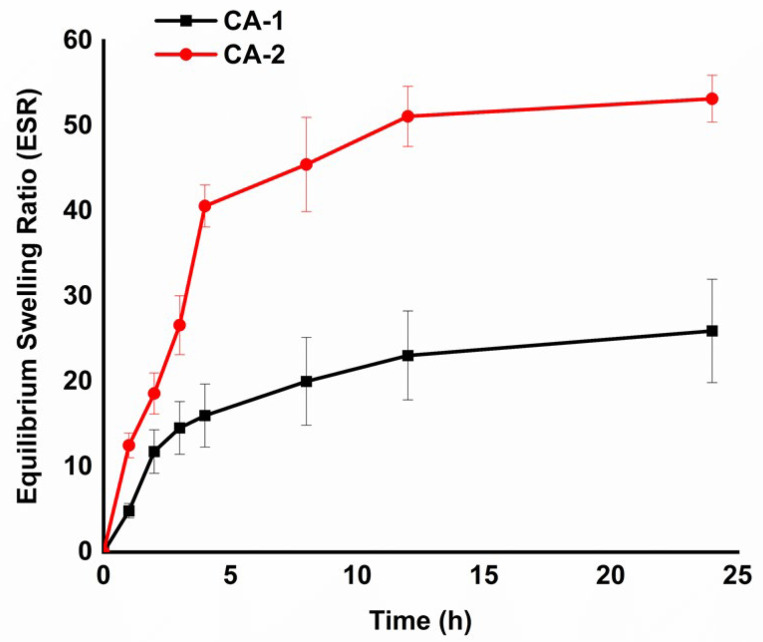
Equilibrium swelling capability of hydrogels in PBS (7.4).

The swelling ratio of hydrogels has a considerable impact on drug-loading efficiency. The swelling characteristics of the hydrogels were assessed using a gravimetric approach. The equilibrium swelling behavior is presented in Figure 4, which demonstrates that hydrogel CA-1 and CA-2 swell dynamically during the initial 4 h, indicating a 12, and 40 times rise in mass as in comparison to their original mass, respectively. The hydrogels gradually captivated water during the initial period of swelling and essentially attained their swelling equilibrium by 24 h. The CMC/Alg-derived hydrogels showed higher swelling behavior than Alg/PEG-derived hydrogels in the previous report [13].

Interestingly, both the carbohydrate polymers have COOH functional groups in their respective backbones, leading to a higher ESR. At a pH of 7.4, the Alg and CMC chains experience deprotonation, resulting in the formation of negatively charged –COO^−1^ anions. This process leads to the formation of repulsive forces within the polymer networks. These anionic groups could induce more hydrophilicity and hydrogel networks expanded more effectively [37]. The results suggest that the CMC/Alg-derived hydrogels will be beneficial for drug delivery application due to the higher swelling properties.

### 2.5. Morphology of Hydrogels

**Figure 5:**
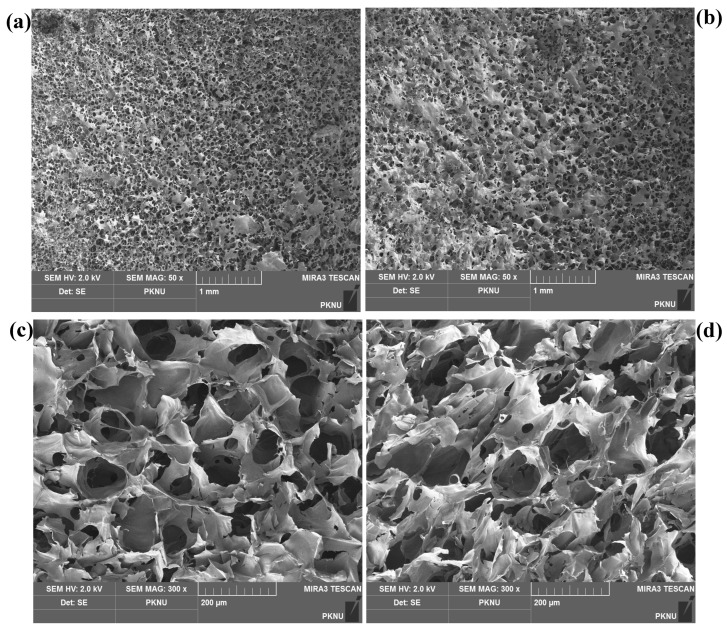
FE-SEM images of surface morphology (**a**,**b**) and cross-sectional area (**c**,**d**) of CA-1 and CA-2 hydrogels, respectively.

The CMC/Alg-derived hydrogels exhibited porous morphologies, which have the potential to provide advantages in various biological applications, including drug delivery and tissue engineering. The highly porous morphologies observed in the injectable hydrogels could be attributed to the in situ formation of micro-bubbles within the networks induced by the generation of N_2_ gas during the IEDDA click reaction [14]. Indeed, the IEDDA click reaction, involving the interaction between methyl-tetrazine (diene: electron-deficient species) and Nb (dienophile: electron-rich species), demonstrated high efficacy under physiological conditions. This reaction resulted in the formation of covalent cross-linking bonds and the release of N_2_ gas as a by-product. The porous morphologies of the hydrogels were visualized using FE-SEM after they underwent lyophilization. It is notable that more and smaller holes are observed on the surface of the hydrogels at the higher molar concentration of Alg–mTz (see Figure 5a,b).

This phenomenon can be attributed to that a greater number of small bubbles were formed in the denser network resulted from the higher IEDDA cross-linking reaction. Meanwhile, the internal structure of all the formulated hydrogels exhibited a high level of porosity, as depicted in Figure 5c,d. Well-connected pores are evident within the hydrogel system when a higher molar feed ratio of Alg–mTz is employed (see Figure 5c). In comparison, more open pores are observed with the lower molar feed ratio of Alg–mTz, as illustrated in Figure 5d. Porosity in injectable CMC/Alg-based hydrogels may play a crucial role in processes such as swelling, drug release, and efficient oxygen supply within the 3D microenvironment.

### 2.6. In Vitro ROS Detection and Photodynamic Effect

The production of ROS resulting from the interaction between NIR light and the ICG molecule was evaluated using DPBF as a probe. DPBF is a fluorescent molecule that exhibits a highly specific reaction with ROS, including singlet oxygen and radicals such as hydroxy, alkoxy, alkyloxy, and alkylperoxy. When exposed to ROS, DPBF undergoes rapid oxidation, leading to the formation of 1,2-dibenzoylbenzene or o-benzoylbenzophenone, which results in a decrease in the fluorescence intensity of DPBF. The fluorescence intensities exhibited consistent changes in correlation with both the duration of irradiation (5–15 min) and the power density of NIR light (1–2 W/cm^2^).

**Figure 6:**
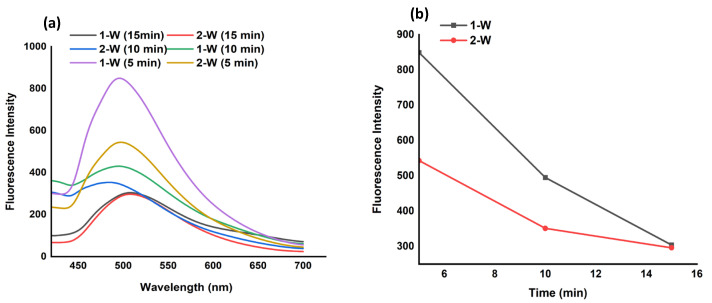
Photodynamic performance of formulated hydrogels (**a**) change in fluorescence intensities of DPBF as a function of ROS production under 1–2 W power NIR light for 5–15 min (**b**) The degradation of DPBF with CA-1 hydrogel at 1–2 W NIR irradiation.

The ICG-loaded hydrogels (CA-1) exhibited its maximum fluorescence intensity when exposed to 1 W NIR light for a duration of 5 min. However, a significant reduction in fluorescence intensities was observed when subjecting the hydrogel to NIR radiation with the higher power density, as illustrated in Figure 6a. The DPBF fluorescent intensities were also reduced with prolonged irradiation time (10–15 min), as depicted in Figure 6b. The porous structure of the CMC/Alg-derived hydrogels might play a crucial role in contributing to their high photothermal and photodynamic performance. The higher porosity facilitates multiple light scattering and reflection, leading to enhanced capture of the incident light and, consequently, improved light absorption [38].

### 2.7. Cytocompatibility Analysis

The assessment of cytocompatibility is a crucial factor when considering hydrogels that could potentially impart harmful effects on biological processes. In this context, an in vitro examination of the cytotoxicity of CMC–Nb and Alg–mTz was conducted using HEK-293 cells, employing the water-soluble tetrazolium salt (WST) assay. To assess cytotoxicity, HEK-293 cells were subjected to varying concentrations of CMC–Nb and Alg–mTz for a duration of 24 h, after which cell viability was ascertained under controlled light conditions.

**Figure 7:**
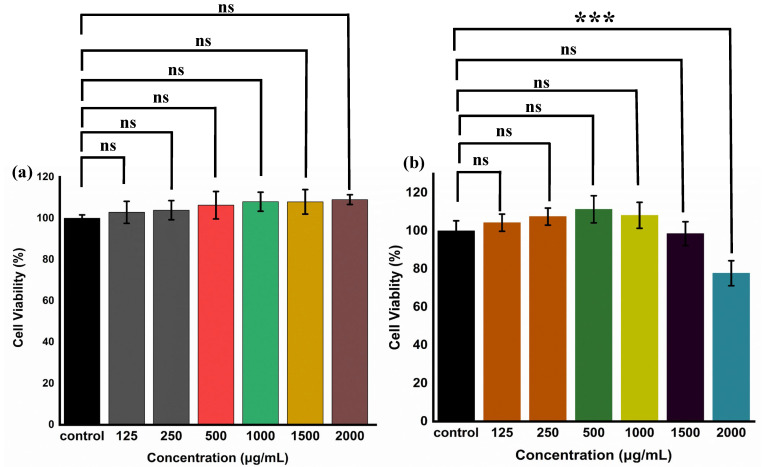
The In vitro cytocompatibility of precursors in tested HEK-293 cells. (**a**) cytocompatibility of CMC–Nb (ns = non-significant) and (**b**) cytocompatibility of Alg–mTz (*** = very significant (*p* = 0.001868)) for 2000 µg/mL Alg–mTz. All samples were treated for 24 h. Error bars showing standard deviation (*n* = 4).

The results of the cytotoxicity assessments revealed that the presence of CMC–Nb and Alg–mTz did not exert a significant impact on the proliferation of HEK-293 cells. Notably, CMC–Nb exhibited remarkable biocompatibility and bio-safety, even at the highest concentration tested, which was 2000 µg/mL, with a striking 98% cell viability, as presented in Figure 7a. These findings highlight the concept that the inclusion of Nb moieties within the structure of CMC does not detrimentally affect its biocompatibility, thereby establishing CMC–Nb as a suitable precursor for the production of bio-orthogonal hydrogels [13]. Similarly, Alg–mTz displayed cell viabilities reaching approximately 80% following exposure to HEK-293 cells at the maximal concentration of 2000 µg/mL, as depicted in Figure 7b. This observation further highlights the biocompatibility of these precursor materials, which not only permits effective cell proliferation but also demonstrates cell viability levels comparable to those of control groups, thereby affirming their suitability for downstream applications.

### 2.8. Live/Dead Assay

**Figure 8:**
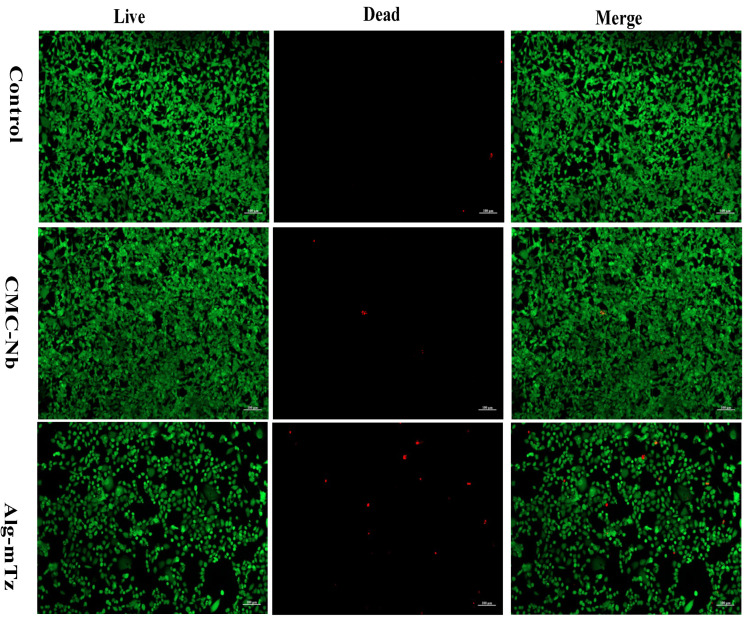
Confocal laser scanning micrographs showing cytocompatibility of control mdia, CMC–Nb, and Alg–mTz in HEK-293 cells. Cell viabilities were determined by calcein-AM/ethidium homodimer-1 assay (live/dead assay) Green color represents live cells, whereas, red color represents dead cells. Scale bars showing 100 μm. (For interpretation of the references to color in this figure legend, the reader is referred to the web version of this article).

The live/dead assay, utilizing calcein-AM and ethidium bromide, was carried out to confirm further the biocompatibility of the precursors. In the confocal images, viable cells were prominently displayed in green, while deceased cells were marked in red (see Figure 8). These findings revealed that there was no significant variance in the count of live and dead cells when exposed to the control, CMC–Nb, and Alg–mTz.

### 2.9. The Photothermal Effect under NIR Irradiation and Photothermal Conversion Efficiency of Hydrogels

**Figure 9:**
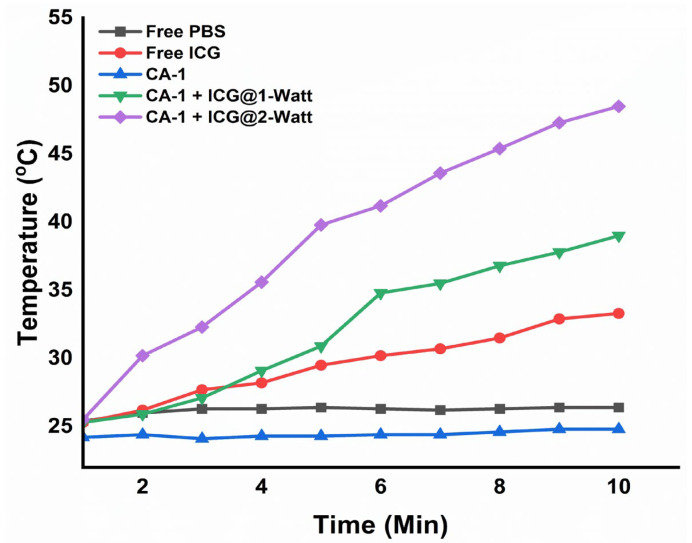
Photothermal effect of free PBS, free ICG, and formulated hydrogels under different intensities of NIR irradiation.

To assess the potential for hyperthermia induction, NIR laser light was employed to measure temperature elevations (ΔT) in both the CMC/Alg-derived hydrogels and their respective solutions. As the duration of NIR light exposure increased incrementally from 0 to 10 min, a corresponding temperature rise was observed, reflecting also the increased power of the NIR light source (see Figure 9).

An examination of thermal images (Figure S2) revealed that hydrogel CA-1 and the free PBS solution exhibited negligible temperature fluctuations following 10 min of laser irradiation. In contrast, free ICG and ICG-loaded hydrogels exhibited substantial temperature variations in direct correlation with the increasing intensity of the laser irradiation. It is worth noting that there exists a directly proportional relationship between laser intensity and the resulting temperature elevation.

For instance, when exposed to a 1 W NIR laser, free ICG and CA-1 + ICG experienced temperature elevations of 33 °C and 38.5 °C, respectively, over 10 min. A doubling of the laser intensity to 2 W escalated the temperature to 43 °C and 48.5 °C for free ICG and CA-1 + ICG, respectively, in the same time frame. These findings underscore the clear dependence of the temperature elevation of the ICG-loaded hydrogels on NIR laser intensity, a crucial characteristic for inducing hyperthermal effects with the potential to ablate cancer cells [20]. The photothermal conversion efficiency (η) of the ICG-loaded (1 mg/mL) hydrogels irradiated under 808 nm laser (2 W/cm^−2^) for 10 min. was calculated to be 34.6% [39].

### 2.10. Anti-Cancer Effect of Hydrogels

**Figure 10:**
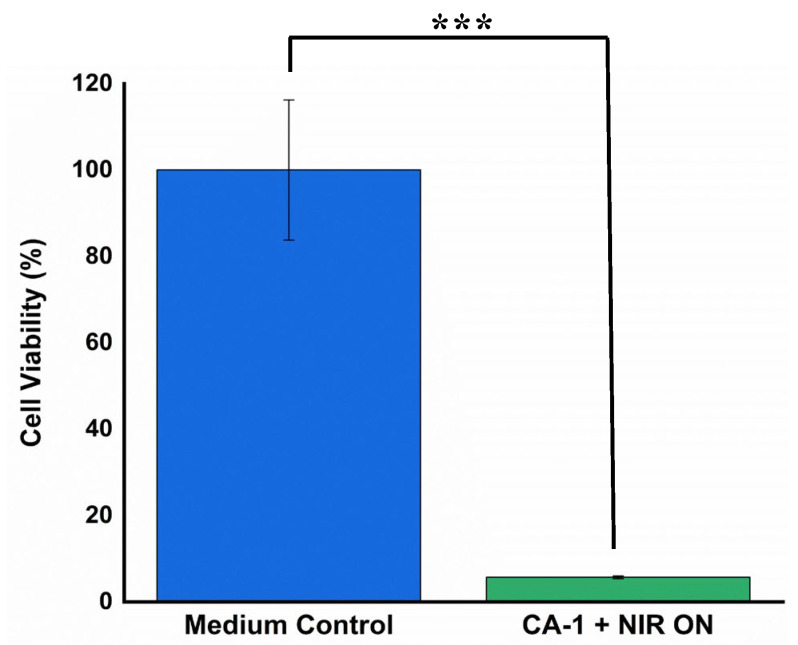
Anti-cancer treatment against HeLa cancer cells with/without NIR irradiation (2 W power, 180 s) (*** = very significant (*p* = 0.0004683)). Error bars showing standard deviation (*n* = 4).

To investigate the NIR light-induced PDT/PTT and its associated anti-tumor efficacy, CA-1 was suspended in a culture medium containing HeLa cells and subjected to NIR irradiation (2 W, 180 s). The viability of HeLa cells, both with/without NIR treatment, after incubation with the hydrogels, is graphically represented in Figure 10. Anti-tumor effect was not significantly observed without NIR irradiation due to the absence of ROS and heat generation. By contrast, the high anti-tumor efficacy (i.e, 6% cell viability) of CMC/Alg-derived hydrogels under NIR exposure is indicative of their potential for photothermal and photodynamic therapeutic applications, primarily attributed to the rapid ROS generation and heat within the hydrogel matrix.

## 3. Conclusions

The ICG-loaded CMC/Alg hydrogel system was developed for combined PDT and PTT applications. The injectable hydrogels were fabricated through a process of CMC–Alg conjugation, employing CMC functionalized with Nb groups and Alg modified with mTz groups, which reacted together quickly through the IEDDA click chemistry. Varying the molar ratio of Alg–mTz within the hydrogel formulations led to distinct mechanical properties, with storage modulus (G′) ranging from approximately 492 to 270 Pa. The hydrogels exhibited significant swelling capacity and high porosity. Upon exposure to different NIR irradiation (1–2 W/cm^2^) for prolonged time (5–15 min), the ICG-loaded hydrogel (CA-1) demonstrated a rapid increase in temperature. The generation of ROS in the hydrogels was also confirmed after NIR exposure by the DPBF assay. Cytotoxicity assessments on HEK-293 cells indicated that both precursor materials, CMC–Nb and Alg–mTz, were non-toxic, underscoring their good compatibility with the tested cell line. Furthermore, CA-1 hydrogels exhibited notable anti-tumor activity when exposed to NIR radiation, particularly against HeLa cancer cells. Consequently, it is believed that these hydrogels hold great promise for potential applications in combined PTT and PDT.

## 4. Materials and Methods

### 4.1. Materials

Sodium alginate (*M*_w_ = 120–190 kDa), CMC (*M*_w_ ~ 276 kDa), 5-norbornene-2-methylamine, 1-ethyl-3-(3-dimethylaminopropyl)-carbodiimide hydrochloride (EDC. HCl, 99%), and ICG were procured from Tokyo Chemical Industries (TCI, Tokyo, Japan). Methyl tetrazine amine HCl salt was purchased from Conju-Probe, LLC, San Diego, CA, USA. N-hydroxysuccinamide (NHS 98%) was acquired from Sigma Aldrich, St. Louis, MO, USA. All other reagents were of analytical quality and used without further processing.

### 4.2. Instruments

The analysis of 1H-NMR spectra was conducted with an NMR instrument, specifically the JNM ECZ-400 model from JEOL. An Agilent CARY 640 spectrometer (Agilent Technologies, Santa Clara, CA, USA) was used to acquire FTIR spectra. The surface and internal structures of CMC/Alg hydrogels were examined by means of field emission scanning electron microscopy (FE-SEM, MIRA 3 system TESCAN). A UV-Vis spectrophotometer (Optizen POP, Optizen, Daejeon, Republic of Korea) was utilized to measure UV-Vis spectra in the range of 4000–800 cm^−1^. The mechanical properties of the scaffolds were assessed using a hybrid rheometer (DHR-2, TA-instrument, New Castle, DE, USA).

### 4.3. Polymer Conjugation for the Development of Hydrogels

#### 4.3.1. Coupling of Alg with mTz

Alg–mTz was synthesized following the previously established protocol with minor adjustments, as illustrated in Scheme 2A [37]. Firstly, 1000 mg of sodium alginate (0.0065 mmol) was dissolved in 100 mL of deionized (DI) H_2_O overnight to make a homogenous solution. EDC. HCl (193 mg, 1.005 mmol) and NHS (174 mg, 1.508 mmol) pre-dissolved in DI H_2_O and DMSO respectively, were added into the Alg solution and allowed to react for one hour. Afterward, mTz (239 mg, 1.006 mmol) pre-dissolved in DMSO (2 mL) was introduced slowly into the reaction vessel.

The solution was agitated for 24 h at room temperature (RT). An excessive amount of acetone (3 × 100 mL) was added to quench the reaction, resulting in the formation of a pink-colored solid product. The product was dried and re-dissolved in DI water. Then the product was continuously dialyzed through dialysis tubing (MWCO = 14 kDa) for 72 h. Finally, the conjugated material underwent a 48-h freeze-drying process, leading to Alg–mTz with a good yield (65%). The conjugation of Alg with mTz was clarified through ^1^H-NMR spectroscopic analysis.

#### 4.3.2. Conjugation of CMC with Norbornene Amine

CMC underwent chemical modification with a norbornene moiety via an amidation process using an EDC coupling reaction, in line with the previously published work [13]. The process, as depicted in Scheme 2B, commenced with the dissolution of 1000 mg of Na–CMC (0.0036 mmol) in 100 mL of DI H_2_O at RT overnight, resulting in a homogenous solution (1% *w*/*w*). In the subsequent step, EDC. HCl (580 mg, 3.04 mmol) and NHS (340 mg, 3.04 mmol) were introduced into the CMC solution to activate the COOH functional groups along the CMC backbone. The reaction was stirred at RT for 2 h. After completing the activation step, 370 µL (3.04 mmol) of 5-norbornene-2-methylamine was added incrementally to the reaction mixture, which was then stirred at RT for 24 h. The product was precipitated by employing 3 × 100 mL acetone. The resulting white fluffy product was obtained through vacuum filtration and subsequently dried in a vacuum oven. The product was then re-dissolved in deionized water to attain a 1% concentration (*w*/*v*) and subjected to dialysis using a 14 kDa MWCO membrane tubing for 72 h. The final yield of CMC with norbornene (CMC–Nb), which surpassed 80%, was achieved through lyophilization. The confirmation of CMC modification with norbornene amine was verified using ^1^H-NMR spectroscopy.

### 4.4. Hydrogel Fabrication

To prepare CMC/Alg-based hydrogels, a vial was charged with 200 µL of CMC–Nb (2.5% *w*/*v*) in DI water and subjected to stirring until a uniform solution was achieved. Simultaneously, two weight % ratios of Alg–mTz (200 µL) (as shown in Table 1) were dissolved in DI water, contingent upon different molar ratios of Nb/Tz (1:1.70 and 1/0.85). Both CMC–Nb and Alg–mTz solutions were mixed and subjected to vortexing for 20 s to promote the formation of a homogeneous phase, facilitating hydrogel fabrication as depicted in Scheme 2C.

### 4.5. Characterization

#### 4.5.1. Rheological Analyses of CMC/Alg Hydrogels

The mechanical properties of CMC/Alg-based hydrogels were carried out by dynamic oscillation of two parallel plates to determine the storage (G′) and loss (G″) moduli respectively. In the frequency sweep test, a constant strain (1%) was applied with varying the angular frequency from 0–100 rad/s, while the dynamic oscillatory strain amplitude sweep experiments were measured with the applied strain range (0.1 to 10,000%) keeping the angular frequency constant at 10 rad/s.

#### 4.5.2. Swelling Properties

The ability of hydrogels to swell was evaluated using a gravimetric approach. Lyophilized hydrogels were placed in a sufficient quantity of pH 7.4 PBS solution at physiological temperature. The weight of the swollen hydrogels was measured at specific time intervals until a stable weight was reached [40]. This experiment was repeated three times for each hydrogel. The equilibrium swelling ratio (ESR) was calculated using the following equation.

ESR% = (*M_s_* − *M_d_/M_d_*) × 100 (1)

In the equation, “*M_s_*” represents the mass of the swollen hydrogels, and “*M_d_*” represents the mass of the lyophilized hydrogels.

#### 4.5.3. The Structural Characteristics of CMC/Alg Hydrogels

FE-SEM was employed to examine the surface and interior structures of the fabricated hydrogels. Prior to analysis, the hydrogel samples were lyophilized and vertically sliced in liquid nitrogen.

#### 4.5.4. In Vitro ROS Generation

The in vitro ROS generation ability of hydrogels (CA-1) was assessed by using 1,3-diphenylisobenzofuran (DPBF) assays. To conduct the experiment, 10 µL of a DPBF solution (3.7 mmol., in DMSO) was introduced into a quartz cuvette containing CMC/Alg hydrogels loaded with 20 µg of ICG. NIR light exposure was performed for 5–15 min at power levels of 1 W and/or 2 W. The fluorescent intensities of the samples were measured in the wavelength range of 420–700 nm, with excitation at 410 nm.

#### 4.5.5. In Vitro Cytocompatibility Analysis of the Precursors and Hydrogels

The cytocompatibility of CMC–Nb, Alg–mTz, and CMC/Alg hydrogels was evaluated using the WST assay (EZ-cytox, Seoul, Republic of Korea) with human embryonic kidney cell line (HEK-293). The cells were cultured in DMEM supplemented with 10% fetal bovine serum (FBS) and 1% antibiotic–antimycotic solution in an incubator at 37 °C with 5% CO_2_ for 24 h, then seeded in a 48-well plate at a density of 10^4^ cells per well. Subsequently, the culture medium was aspirated and replaced with a fresh culture medium containing CMC–Nb (125–2000 µg/mL) and Alg–mTz (125–2000 µg/mL). After 24 h, the cells were rinsed twice with PBS (100 µL). Next, a fresh medium with the WST assay solution (10 μL) was added to each well. Cell viability was determined by measuring the optical density at a wavelength of 450 nm using a microplate reader.

#### 4.5.6. Fluorescence-Based Live/Dead Assay

Cellular viability and cell death assays for control, CMC–Nb and Alg–mTz (equivalent to 20 µg of ICG) were conducted on HEK-293 cells. After the treatments mentioned above, the cells were stained with calcein-AM to distinguish viable cells (appearing green) and then further stained with ethidium bromide to detect non-viable cells (appearing red) using a fluorescence microscopy technique.

#### 4.5.7. In Vitro Photothermal Effect and Photothermal Conversion Efficiency of Hydrogels

The photothermal test consisted of exposing free PBS, free ICG (1 mg/mL in PBS), and the ICG-loaded hydrogel (CA-1) to NIR laser irradiation, with power settings at both 2 W/cm^2^ and 1 W/cm^2^ for a duration of 10 min. Temperature fluctuations in the samples were monitored using a thermometer every minute over the course of 10 min.

The photothermal conversion efficiency η of Alg/CMC hydrogels was calculated according to the following equations:

(2) $$\eta =\frac{hA\left(T_{max}{\;-\;T}_{surr}\right){\;-\;Q}_{Dis}}{I\left({1\;-\;10}^{-A\lambda }\right)}$$

(3) $$Q_{Dis}=h_{0}A\left(T_{max}\;-\;{\;T}_{surr}\right)$$

where *h* is heat transfer coefficient, *A* is surface area of the container, *T_surr_* is the ambient temperature, *T_max_* is the highest temperature of the sample under NIR irradiation 808 nm laser, *I* is the power density of laser (2 W/cm^2^), *A_λ_* is the is the absorbance of ICG at 808 nm, and *Q_Dis_* is the heat loss of the light absorbed the quartz sample cell [39].

#### 4.5.8. Anti-Cancer Effect of the ICG-Loaded Hydrogels

The anti-cancer potential of the ICG-entrapped hydrogels was evaluated using a WST assay with a HeLa cell line. The cells were cultured and seeded in a 48-well plate. The existing culture medium was replaced with a fresh medium containing PBS (pH 7.4). Subsequently, the cells were exposed to CA-1 (by placing hydrogels on the cell culture medium) for 48 h, followed by treatment with NIR irradiation (808 nm, 3 min, 2-W power) [41].

#### 4.5.9. Statistical Analysis

All physiochemical investigations were conducted in triplicate, while cell culture assays were performed with four replicates. The data are presented as the mean ± standard deviation the student’s *t*-test was used to evaluate the significance. The statistically significant (*p* < 0.05) and very significant (*p* < 0.001) were used to consider the difference between two results.

## Figures and Tables

**Table 1:** Molar feed ratios of CMC/Alg hydrogels.

Hydrogel Code	CMC–Nb:Alg–mTz (% *w*/*v*)	Nb:Tz mmol
CA-1	2.5:2.5	1:1.70
CA-2	2.5:1.25	1:0.85

## Data Availability

The data presented in this study are openly available in article.

## Supplementary Materials

The following supporting information can be downloaded at: https://www.mdpi.com/article/10.3390/gels9120961/s1, Figure S1: Photographic images of fabricated hydrogels derived from bio-conjugated polysaccharide; Figure S2: Photothermal effect of NIR irradiation on hydrogels.

## Abbreviations

**Abbreviation**	**Description**
Alg	Alginate
CMC	Carboxymethyl cellulose
Alg–mTz	Alginate methyl tetrazine
CMC–Nb	Carboxymethyl cellulose norbornene
DOX	Doxorubicin
DPBF	1,3-diphenylisobenzofuran
EDC.HCl	1-ethyl-3-(3-dimethylaminopropyl)-carbodiimide hydrochloride
FBS	Fetal bovine serum
G′	Shear storage modulus
G′’	Shear loss modulus
HEK-293	Human embryonic kidney-293
ICG	Indocyanine green
IEDDA	Inverse electron-demand Diels–Alder
NHS	N-hydroxysuccinamide
NIR	Near Infrared
LVR	Linear viscoelastic region
PTT	Photothermal therapy
PDT	Photodynamic therapy
ROS	Reactive oxygen species
WST	water-soluble tetrazolium salt
