# Supplementary material for: Facile Fabrication of NIR-Responsive Alginate/CMC Hydrogels Derived through IEDDA Click Chemistry for Photothermal–Photodynamic Anti-Tumor Therapy
Source: Gels. 2023 Dec 7;9(12):961. doi: 10.3390/gels9120961 (PMC10742702; doi:10.3390/gels9120961)
Supplement: Supplementary file 1 [file gels-09-00961-s001.zip › gels-2756428-supplementary.pdf]

Supplementary Materials

Facile Fabrication of NIR-Responsive Alginate/CMC Hydrogels Derived through IEDDA Click Chemistry for Photothermal–Photodynamic Anti-Tumor Therapy

Ali Rizwan [1], Israr Ali [1], Sung-Han Jo [2], Trung Thang Vu [1], Yeong-Soon Gal [3], Yong Hyun Kim [1,4], Sang-Hyug Park [2,*] and Kwon Taek Lim [4,5,*]

[1] Department of Smart Green Technology Engineering, Pukyong National University, Busan 48513, Republic of Korea; arizwan92@outlook.com (A.R.); israrchem@gmail.com (I.A.); vutrungthang29@gmail.com (T.T.V.); yhkim113@pknu.ac.kr (Y.H.K.)
[2] Industry 4.0 Convergence Bionics Engineering, Pukyong National University, Busan 48513, Republic of Korea; josunghan91@gmail.com
[3] Department of Fire Safety, Kyungil University, Gyeongsan 38428, Republic of Korea; ysgal@kiu.ac.kr
[4] Major of Display Semiconductor Engineering, Pukyong National University, Busan 48513, Republic of Korea
[5] Institute of Display Semiconductor Technology, Pukyong National University, Busan 48513, Republic of Korea
* Correspondence: shpark1@pknu.ac.kr (S.-H.P.); ktlim@pknu.ac.kr (K.T.L.); Tel.: +82-51-629-6406 (K.T.L.)

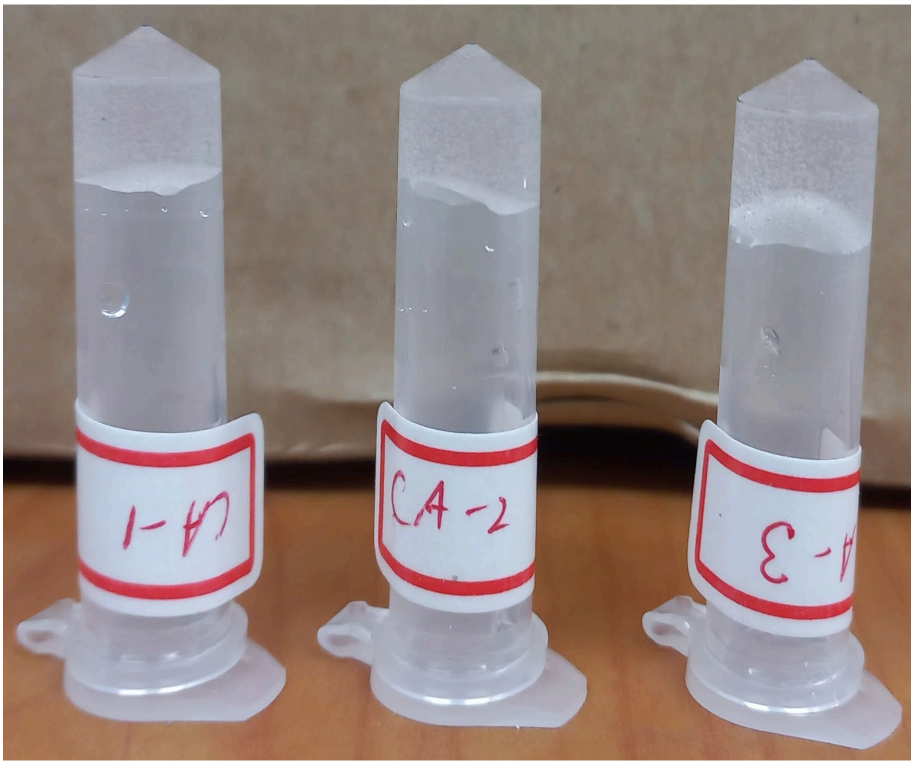

Figure S1. Photographic images of fabricated hydrogels derived from bio-conjugated polysaccharide.

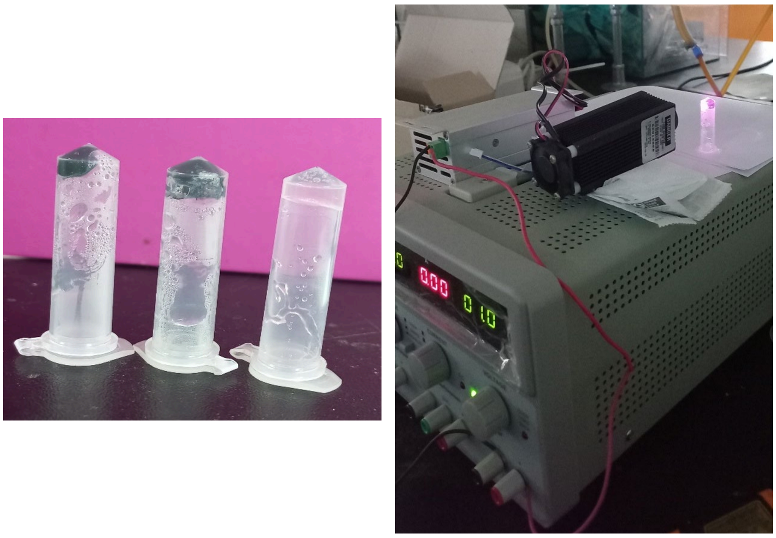

Figure S2. Photothermal effect of NIR irradiation on hydrogels.
